# Hemorrhagic Diathesis in *Borrelia recurrentis* Infection Imported to Germany

**DOI:** 10.3201/eid2205.151557

**Published:** 2016-05

**Authors:** Christian Keller, Malte Zumblick, Katrin Streubel, Markus Eickmann, Daniela Müller, Martina Kerwat, Stephan Becker, Thomas Gress

**Affiliations:** University Hospital Marburg, Marburg, Germany

**Keywords:** Borrelia recurrentis, louseborne relapsing fever, Somalia, ceftriaxone, doxycycline, Germany, vector-borne infections, lice, refugees, bacteria

**To the Editor:** Relapsing fevers are paroxysmal bloodstream infections caused by spirochetes of the genus *Borrelia*. Louseborne relapsing fever (LBRF; i.e., epidemic relapsing fever) is caused by *B. recurrentis* and transmitted by the human body louse (*Pediculus humanus*). Soft ticks of the Argasidae family (e.g., *Ornithodorus moubata*) are vectors for tickborne relapsing fever (TBRF) borreliae, which encompass several human-pathogenic species. In Europe, LBRF was epidemic in the early 20th century but is now rarely seen. We report an infection with *B. recurrentis* imported to Germany by a Somalian refugee who had high fever and hemoptysis and describe the process of molecular diagnosis.

In August 2015, an 18-year-old man sought asylum in Germany after travel through Somalia, Ethiopia, Sudan, Libya, and Italy. He reported general weakness and fever while in Libya, ≈16 days before seeking care, and started coughing up blood after arriving in Italy. At hospital admission in Germany, he had a temperature up to 40.4°C, cough, and hemoptysis; his suspected diagnosis was tuberculosis. No ectoparasites were reported or found on physical examination. Abnormal laboratory findings included relative neutrophilia (91% [reference 39%–77%]), thrombocytopenia (platelets 112 × 10³/μL [reference 160–385 × 10³/μL]), and prolonged activated partial thromboplastin time (APTT) (Figure, panel A). Because of highly elevated levels of C-reactive protein (250 mg/L [reference <5 mg/L]) and procalcitonin (16.4 µg/L [reference <0.5 µg/L]), the patient was treated with ceftriaxone (2g/d intravenously), metronidazole (500 mg/d intravenously), and paracetamol (acetaminophen). Repeated examinations of Giemsa-stained thick and thin blood slides were negative for malaria parasites. Blood cultures, tests for tuberculosis, and PCRs for Rift Valley fever, yellow fever, dengue, and chikungunya viruses also were negative. With antimicrobial therapy, the patient’s fever declined within 12 hours, but platelet counts further decreased and APTT continued to increase (Figure, panel A).

**Figure:**
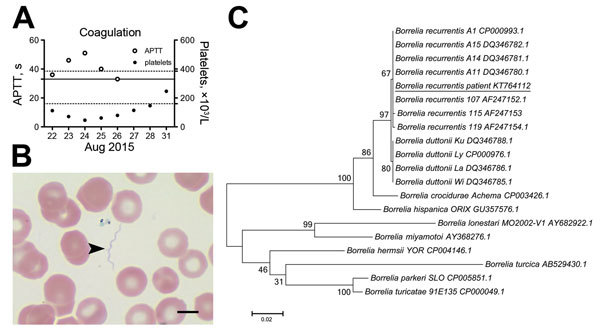
Laboratory findings of hemorrhagic diathesis in an 18-year-old Somalian refugee to Germany with *Borrelia recurrentis* infection, August 2015. A) Time course of coagulation parameters (thrombocytopenia and prolongation of activated partial thromboplastin time [APTT]). B) Extracellular spirochetes demonstrated by light microscopy (arrowhead). Representative image detail from thin blood smear, Giemsa stain. Scale bar indicates .5 μm. C) Molecular phylogenetic analysis of *B. recurrentis* detected in patient blood. Multiple alignment of complete *glpQ* sequence (1,002 bp) with published reference sequences was performed by using BioEdit 7.0.5.3 software (Ibis Biosciences, Carlsbad, CA, USA) and analyzed by MEGA6.06 (http://www.megasoftware.net). The evolutionary history was inferred by using maximum-likelihood method based on the Tamura-Nei model. Bootstrap values are shown at the node of branches (1,000 bootstrap replications). The complete *glpQ* sequence was deposited in GenBank under accession no. KT764112. Scale bar indicates nucleotide substitutions per site.

The patient’s symptoms and travel history raised suspicion of a spirochete infection. A plasma sample from his second day in the hospital tested positive for *Borrelia* spp. 16S DNA by real-time PCR (*1*). Retrospective microscopy revealed a low number of extracellular spirochetes in thin blood smears (Figure, panel B). The antimicrobial regimen was changed to doxycycline (100 mg 2×/d) on day 7 after admission and, because species identification had not been completed, continued for 10 days. No signs of a Jarisch-Herxheimer reaction were seen. During days 4–9 after admission, APTT, platelet counts (Figure, panel A), and C-reactive protein values returned to normal, and the patient was discharged.

For species identification, we amplified the entire coding sequence of *glpQ* (glycerophosphodiester phosphodiesterase) with newly designed primers (Technical Appendix). The amplicon was 100% (1,002/1,002 bp) identical to *B. recurrentis* A1 (GenBank accession no. CP000993.1) and 99% identical (999/1,002 bp) to *B. duttonii* Ly (GenBank accession no. CP000976.1). A phylogenetic analysis that included 7 published *glpQ* sequences from *B. recurrentis* and 4 from *B. duttonii* suggested that the detected pathogen clustered with *B. recurrentis* and not *B. duttonii* (Figure, panel C).

Borreliae have been recognized as a frequent cause of febrile infections in West and East Africa (*2*). Data on the incidence in immigrants are not available, but the recent increase in asylum seekers from East Africa arriving in Central Europe has increased attention of *Borrelia* as a pathogen to be included in differential diagnoses of febrile infections (*3*,*4*). Because symptom onset in the patient we report occurred in Libya, he most likely acquired infection on the African continent, although local transmission in Europe can occur (*4*).

Blood slide examination, which would show spirochetes, is routinely requested to detect *Plasmodium* parasites, but its sensitivity in detecting borreliae is strikingly inferior to molecular tools (15%–56%, depending on laboratory conditions) (*1*). Pan-*Borrelia* real-time PCRs enable sensitive detection of DNA in blood samples, followed by sequencing (*1*) or confirmatory PCRs for relapsing fever *Borrelia*–specific genes (e.g., *glpQ*) (*5*,*6*). *B. recurrentis* is genetically highly similar to *B. duttonii,* suggesting it might be a degraded subset of its tickborne counterpart rather than a distinct species (*7*). Yet, phylogeny of whole *glpQ* sequences enables separation of *B. recurrentis* from *B. duttonii* on the basis of distinct single-nucleotide variations. Alternatively, differentiation can be achieved by phylogenetic analysis of concatenated partial 16s, *glpQ* and *flaB* (flagellin) sequences (*5*). Differentiation between TBRF and LBRF is crucial for the correct clinical decision on therapy duration, independent of the antimicrobial substance chosen: at least 7 days of treatment is recommended for TBRF to prevent relapses after early invasion of spirochetes into the central nervous system (*8*), whereas a single-dose regimen is sufficient for LBRF (*9*), although longer treatment courses tend to be used.

In summary, our report emphasizes that LBRF can be complicated by pulmonary hemorrhages associated with impaired platelet and plasmatic coagulation (*10*), which can be mistaken for signs of tuberculosis. Considering the poor hygienic conditions among refugees, LBRF has become an important differential diagnosis in Europe in times of increasing migration.

Technical Appendix Oligonucleotide sequences used for amplification of the *glpQ* gene.
