# Supplementary material for: Hemorrhagic Diathesis in Borrelia recurrentis Infection Imported to Germany
Source: Emerg Infect Dis. 2016 May;22(5):917–9. doi: 10.3201/eid2205.151557 (PMC4861516; doi:10.3201/eid2205.151557)
Supplement: Technical Appendix — Oligonucleotide sequences used for amplification of the glpQ gene. [file 15-1557-Techapp-s1.pdf]

Article DOI http://dx.doi.org/10.32032/eid2205.151557

Hemorrhagic Diathesis in *Borrelia recurrentis* Infection Imported to Germany

Technical Appendix

Technical Appendix Table. Oligonucleotide sequences used for amplification* of the *glpQ* (glycerophosphodiester phosphodiesterase) gene

Primer name	Primer sequence	Annealing temperature
glpQ_seq_F	5′-ATTCATCAAAATATAAgTTATgAgAgg-3′	52°C
glpQ_seq_R	5′-AgATATTCTCCTTATTAAAATTATgg-3′	52°C

*The PCR was run in 40 cycles.
